# Supplementary material for: DNA-RNA hybrids at DSBs interfere with repair by homologous recombination
Source: eLife. 2021 Jul 8;10:e69881. doi: 10.7554/eLife.69881 (PMC8289408; doi:10.7554/eLife.69881)
Supplement: Supplementary file 1. [file elife-69881-supp1.docx]

**Supplementary File 1. Strains used in this study**

| Name | Genotype | Source |
| --- | --- | --- |
| WFLP | *MATa-inc trp1-1 ura3-1 ade2-1 his3-11,15 can1-100*  *leu2Δ::SFA1 met17Δ::GAL-flpH305L::HPHMX6* | (Ortega et al., 2019) |
| WFR1R2 | *MATa-inc trp1-1 ura3-1 ade2-1 his3-11,15 can1-100 leu2Δ::SFA1 met17Δ::GAL-flpH305L::HPHMX6 rnh1Δ::NATNT2 rnh201Δ::KANMX4* | This study |
| WFHPR1 | *MATa-inc trp1-1 ura3-1 ade2-1 his3-11,15 can1-100 leu2Δ::SFA1 met17Δ::GAL-flpH305L::HPHMX6 hpr1Δ::HIS3* | This study |
| WSR-7D | *MATa-inc trp1-1 ura3-1 ade2-1 his3-11,15 can1-100*  *ade3::GAL-HO leu2Δ::SFA1* | (Muñoz-Galván et al., 2013) |
| WSR1R2 | *MATa-inc trp1-1 ura3-1 ade2-1 his3-11,15 can1-100 ade3::GAL-HO leu2Δ::SFA1 rnh1Δ::NATNT2 rnh201Δ::KANMX4* | This study |
| WSHPR1 | *MATa-inc trp1-1 ura3-1 ade2-1 his3-11,15 can1-100 ade3::GAL-HO leu2Δ::SFA1 hpr1Δ::HIS3* | This study |
| GLY | *Mata trp1-1 ura3-1 ade2-1 his3-11,15 can1-100 leu2-3,112 NATNT2::GAL-LYS2* | This study |
| GLFT | *Mata trp1-1 ura3-1 ade2-1 his3-11,15 can1-100 leu2-3,112 NATNT2::GAL-lys2-FRT-T* | This study |
| GLFNT | *Mata trp1-1 ura3-1 ade2-1 his3-11,15 can1-100 leu2-3,112 NATNT2::GAL-lys2-FRT-NT* | This study |
| YBP250 | *Matα trp1-1 ura3-1 ade2-1 his3-11,15 can1-100 leu2-3,112 bar1Δ* | (Moriel-Carretero and Aguilera, 2010) |
| YLY | *Matα trp1-1 ura3-1 ade2-1 his3-11,15 can1-100 leu2-3,112 lys2-3705* | This study |
| DGLFT | *Mata/α trp1-1/trp1-1 ura3-1/ura3-1 ade2-1/ade2-1 his3-11,15/his3-11,15 can1-100/can1-100 leu2-3,112/leu2-3,112 NATNT2::GAL-lys2-FRT-T/lys2-3705* | This study |
| DGLFNT | *Mata/α trp1-1/trp1-1 ura3-1/ura3-1 ade2-1/ade2-1 his3-11,15/his3-11,15 can1-100/can1-100 leu2-3,112/leu2-3,112 NATNT2::GAL-lys2-FRT-NT/lys2-3705* | This study |

Moriel-Carretero, M., and Aguilera, A. (2010). A Postincision-Deficient TFIIH Causes Replication Fork Breakage and Uncovers Alternative Rad51- or Pol32-Mediated Restart Mechanisms. Mol. Cell *37*, 690–701.

Muñoz-Galván, S., Jimeno, S., Rothstein, R., and Aguilera, A. (2013). Histone H3K56 Acetylation, Rad52, and Non-DNA Repair Factors Control Double-Strand Break Repair Choice with the Sister Chromatid. PLoS Genet. *9*, 1–12.

Ortega, P., Gómez-González, B., and Aguilera, A. (2019). Rpd3L and Hda1 histone deacetylases facilitate repair of broken forks by promoting sister chromatid cohesion. Nat. Commun. *10*, 5178.
