# Supplementary material for: DNA-RNA hybrids at DSBs interfere with repair by homologous recombination
Source: eLife. 2021 Jul 8;10:e69881. doi: 10.7554/eLife.69881 (PMC8289408; doi:10.7554/eLife.69881)
Supplement: Supplementary file 2. [file elife-69881-supp2.docx]

**Supplementary File 2. Primers used in this study.**

| Name | Sequence |
| --- | --- |
| FRT_Fw | AAGGACGCAGATGGCAACA |
| FRT_Rv | TCCGATGAAGCCTCCGTTAT |
| PDC1_Fw | GAAGGTATGAGATGGGCTGGTAA |
| PDC1_Rv | CCTTGATACGAGCGTAACCATCA |
| 5FRT_Fw | AGGCAATGGTGGCTCATGTT |
| 5FRT_Rv | AAGTGCAAAGAATCACAAGAATGG |
| 3K_Fw | CAGGGCCAAGGATGAAGAAG |
| 3K_Rv | GTACCATAGGTGATACCTGCCTTT |
| 2.5K_Fw | TGAACCTAATAGTGCCGAAGGA |
| 2.5K_Rv | GGAGCCCAGAAATCCTGTGA |
| Leu Up 2000 | GTTCCACTTCCAGATGAGGC |
| Leu Lo 2000 | TTAGCAAATTGTGGCTTGA |
| ACT1_Fw | TCCCAGGTATTGCCGAAAGA |
| ACT1_RV | TCATGGAAGATGGAGCCAAAG |
| LYSFRTT_Fw | CAACAATTAATGTGTTTGTTACCGGTGTCACAGGATTTCTGGGCTCCGAAGTTCCTATACTTTCTAGAGAATAGG |
| LYSFRTT_Rv | CTTGGCCCTGACGTGGGCAAACACTTTGAAACTGTAGTTCTTTGGAGAACGAAGTTCCTATTCGGAAGTTCC |
| LYSFRTNT_Fw | GAAAAACAACAATTAATGTGTTTGTTACCGGTGTCACAGGATTTCTGGGCTCCGAAGTTCCTATTCGGAAGTTCC |
| LYSFRTNT_Rv | CTTGGCCCTGACGTGGGCAAACACTTTGAAACTGTAGTTCTTTGGAGAACAAGTTCCTATACTTTCTAGAGAATAGG |
| LYS2_2953-72_Fw | GATCTACATCCTTGCAGATTTGTTTTAGAGCTAG |
| LYS2_2953-72_Rv | CATGCTCTAAAACAACAAATCTGCAAGGATGTA |
| LYS2_3700-3719_Fw | GATCGCCAATTCATTTTCTTTGGGGTTTTAGAGCTAG |
| LYS2_3700-3719_Rv | CTAGCTCTAAAACCCCAAAGAAAATGAATTGGC |
| FLP_BamHI_Fw | ACGTGTCATAGGATCCGGTCGACATGCCACAATTTG |
| FLP_Rv | CTAAAGGGAACAAAAGCTGGAGC |
